# Role of Changes in Driving Pressure and Mechanical Power in Predicting Mortality in Patients with Acute Respiratory Distress Syndrome

**DOI:** 10.3390/diagnostics13071226

**Published:** 2023-03-24

**Authors:** Huang-Pin Wu, Shaw-Woei Leu, Shih-Wei Lin, Chen-Yiu Hung, Ning-Hung Chen, Han-Chung Hu, Chung-Chi Huang, Kuo-Chin Kao

**Affiliations:** 1 Division of Pulmonary, Critical Care and Sleep Medicine, Chang Gung Memorial Hospital, Keelung 204, Taiwan; 2 College of Medicine, Chang Gung University, Taoyuan 333, Taiwan; 3 Department of Thoracic Medicine, Chang Gung Memorial Hospital, Taoyuan 333, Taiwan

**Keywords:** driving pressure, mechanical power, mortality, acute respiratory distress syndrome

## Abstract

Driving pressure (ΔP) and mechanical power (MP) are associated with increased mortality in patients with acute respiratory distress syndrome (ARDS). We aimed to investigate which was better to predict mortality between changes in ΔP and MP. We reanalyzed data from a prospective observational cohort study of patients with ARDS in our hospital. Serial ΔP and MP values were calculated. The factors associated with survival were analyzed. Binary logistic regression showed that age (odds ratio (OR), 1.012; 95% confidence interval (CI), 1.003–1.022), Sequential Organ Failure assessment (SOFA) score (OR, 1.144; 95% CI, 1.086–1.206), trauma (OR, 0.172; 95% CI, 0.035–0.838), ΔP (OR, 1.077; 95% CI, 1.044–1.111), change in ΔP (OR, 1.087; 95% CI, 1.054–1.120), and change in MP (OR, 1.018; 95% CI, 1.006–1.029) were independently associated with 30-day mortality. Change in MP, change in ΔP, and SOFA scores were superior to ΔP in terms of the accuracy of predicting 30-day mortality. In conclusion, calculating change in ΔP is easy for respiratory therapists in clinical practice and may be used to predict mortality in patients with ARDS.

## 1. Introduction

According to the Berlin definitions [1], acute respiratory distress syndrome (ARDS) is diagnosed with four inclusion criteria: (1) an acute onset (<1 week of a known clinical insult or new or worsening respiratory symptoms), (2) respiratory failure not primarily due to hydrostatic edema, (3) bilateral opacities on a chest radiograph (not fully explained by effusions, lobar or lung collapse, or nodules), and (4) ≤300 mmHg of the ratio of arterial partial pressure of oxygen (PaO_2_) to the fraction of inspired oxygen (FiO_2_) with ≥5 cm H_2_O of positive end-expiratory pressure (PEEP) or continuous positive airway pressure. To facilitate the estimation of the ARDS prognosis, the Berlin definition classifies the severity of ARDS into 3 categories: mild (200 mmHg < PaO_2_/FiO_2_ ≤ 300 mmHg), moderate (100 mm Hg < PaO_2_/FiO_2_ ≤ 200 mmHg), and severe (PaO_2_/FiO_2_ ≤ 100 mmHg) with a hospital mortality of around 27% for mild ARDS, 32% for moderate ARDS, and 45% for severe ARDS.

Since 2012, the Surviving Sepsis Campaign has recommended specific mechanical ventilation settings for patients with ARDS. These include (1) low tidal volume (*V*_T_) ventilation with a target of a predicted body weight (PBW) of 6 mL/kg, (2) the use of an upper limit goal for plateau pressures (P_plat_) of 30 cm H_2_O, and (3) a relatively higher PEEP [2,3,4]. Except lung protective strategies, driving pressure (ΔP) and mechanical power (MP) have been found to be associated with mortality in patients with ARDS [5,6,7,8].

ΔP can be represented as the ratio of tidal volume to respiratory system compliance, indicating the lung function observed in patients with ARDS. In a study of total 158 severe ARDS patients receiving extracorporeal membrane oxygenation (ECMO), a cox proportional hazards regression model revealed that the means ΔP from day 1 to 3 of ECMO were independently associated with intensive care unit (ICU) mortality [9]. Further, a secondary analysis of data from 787 ARDS patients enrolled in two independent randomized controlled trials found that cox models showed that ΔP, P_plat_, and compliance were independent factors for day-90 mortality but that PEEP and VT were not associated with death [10].

MP is thought of as the energy transferred from the ventilator to the lungs per unit time. It is associated with several underlying relevant factors. One of them is static compliance, which induces ventilator-induced lung injury (VILI) [11]. It also accounts for the final effect of PEEP, which prevents VILI-associated mechanisms such as the over-distention of the alveoli and the cyclic recruitment/de-recruitment or shear stress of lung parenchyma [12]. VILI increases with an increased respiratory rate (RR) [13]. In experimental rats with mild ARDS, even at a low *V*_T_, high MP promoted ventilator-induced lung injury [14]. From the analysis of databases of the MIMIC-III and eICU, even at a low tidal volume, high MP was associated with in-hospital mortality with a consistent increasing risk of death with MP being higher than 17.0 J/min [15]. Furthermore, deep sedation significantly reduced MP in patients with moderate to severe ARDS, thereby reducing the occurrence of VILI [16]. MP monitoring could predict the 28-day survival rate in patients with moderate to severe ARDS.

We hypothesized that as long as *V*_T_ is maintained at a target of 6 mL/kg PBW, MP would exhibit a better correlation with mortality than ΔP would, and that changes in MP might have better predictive values than changes in ΔP in patients with ARDS. Thus, the aim of this study was to investigate these questions.

## 2. Material and Methods

### 2.1. Procedure

We reanalyzed the data from a prospective observational cohort study of patients with ARDS in our hospital. The study design has been described previously [17]. From September 2012 to September 2015, all patients admitted to the ICU with invasive mechanical ventilation were screened, and those who met the diagnostic criteria for ARDS according to the Berlin definition were enrolled [1]. Patients were distributed in one burn ICU, seven surgical ICUs, and nine medical ICUs. The exclusion criteria were an age of <18 years, referral from other hospitals, death within 3 days of ICU admission, ICU stay or ventilator use for less than 3 days, and incomplete data collection. The study was approved by the Institutional Review Board Ethics Committee of Chang Gung Memorial Hospital, and the need for written informed consent was waived (CGMH IRB no. 202200581B0).

### 2.2. Disease Definitions

ARDS was defined according to the Berlin definition [1]. ARDS was evaluated using chest radiographs obtained after intubation with ventilator support. The severity of ARDS was determined by the ratio of the lowest PaO_2_ and FiO_2_ on the first day of mechanical ventilation and classified as mild (200 mmHg < PaO_2_/FiO_2_ ≤ 300 mmHg), moderate (100 mm Hg < PaO_2_/FiO_2_ ≤ 200 mmHg) or severe (PaO_2_/FiO_2_ ≤ 100 mmHg). Pneumonia was defined as a new abnormal infiltration upon chest radiography with respiratory symptoms or fever. Sepsis and septic shock were defined according to the Sepsis-3 guidelines [18]. Sepsis was defined as a suspected or documented infection with an acute increase (≥2) in the Sequential Organ Failure Assessment (SOFA) score. Disease severity was assessed using the Acute Physiology and Chronic Health Evaluation (APACHE) II score [19]. Patients who survived for at least 30 days after the ICU admission were defined as survivors.

### 2.3. Ventilator Strategy and Intensive Management

Patient care was decided by the in-charge physicians without prespecified protocols. All patients used pressure-controlled ventilation (PCV). Volume-controlled ventilation (VCV) was not used due to a rapid change in the peak pressure, which resulted in the risk of VILI. Generally, the tidal volume was maintained between 4 and 8 mL/kg PBW. PBW was calculated as follows: (a) 50 + 0.91 × (height in centimeters − 152.4) for males, and (b) 45.5 + 0.91 × (height in centimeters − 152.4) for females [11]. Moderate to high levels of PEEP (12–16 cm H_2_O) were applied while keeping the plateau airway pressure as low as 30 cm H_2_O whenever possible. Pulse oxymetry was used to maintain the oxygen saturation (SpO_2_) above 90% or PaO_2_ above 60 mmHg by titrating the lowest possible amount of FiO_2_. The ventilator settings were adjusted 2 h after the first setting. Ventilator weaning and adjustment were performed in regular intervals (every 8 h) as necessary, based on the general weaning guidelines and clinical practice of our respiratory therapy department. Intensive management strategies, including fluid resuscitation, the use of antibiotics and/or antiviral agents, the use of corticosteroids, vasopressor infusion, sedation with or without neuromuscular blocking agents, renal replacement therapy, prone positioning, extracorporeal membranous oxygenation, or other supportive treatments were applied at the discretion of the in-charge physician.

### 2.4. Data Records

The following patient data were recorded within 24 h of enrollment: age, sex, body height, body weight, major diagnosis at admission, risk of ARDS development, APACHE II score, SOFA score, and Charlson Comorbidity Index [20]. ΔP was defined as the difference between P_plat_ and PEEP [21] and was recorded every 8 h per day. The mean values of Δ*P*_insp_ (the change in airway pressure during inspiration), RR, *V*_T_, PEEP, and arterial blood gas were recorded daily from the day of ARDS diagnosis to two days after ARDS diagnosis, where Δ*P*_insp_ was the change in airway pressure during inspiration.

### 2.5. Calculation of MP

MP for pressure-targeted ventilation was calculated every 8 h per day according to the following simplified equation [22,23] using RR, *V*_T_ size (L), Δ*P*_insp_, and PEEP: $\mathrm{MP}\;\left(J/\min\right)=0.098\times \mathrm{RR}\times V_{T}\times \left(\Delta P_{\mathrm{insp}}+\mathrm{PEEP}\right)$. Since the simplified equation for MP had a high correlation with the reference value of the MP (*r*^2^ = 0.981; bias + 0.73 J/min) [22], it allowed the estimation of MP for PCV with acceptable results for most clinical situations and a small bias caused by disregarding the effect of the inspiratory pressure rise time.

### 2.6. Statistical Analyses

Statistical analyses were performed using IBM SPSS Statistics, version 27.0.1 for Mac (IBM Inc., Armonk, NY, USA). Continuous variables were presented as means ± standard deviation. The differences between the two groups were analyzed using a Student’s *t*-test, and the differences between the same group were analyzed using a paired sample *t*-test. Categorical variables were reported as numbers (percentages), and the differences in categorical variables between the groups were compared using a Pearson’s chi-squared test or Fisher’s exact test. Univariate binary logistic regression model analyses were performed to study the association between 30-day mortality and all the variables. Statistically significant variables were entered into a multivariate binary logistic regression model to assess their independent contribution to the outcome. Binary variables included in the model were coded as present or absent. The changes in MP and ΔP were defined as the levels 2 days after ARDS diagnosis minus the levels on the day of ARDS diagnosis. Receiver operating characteristic (ROC) curves of ΔP and the changes in MP and ΔP were drawn. The area under the ROC curve (AUROC) was calculated. A Kaplan–Meier graph was plotted to analyze the probability of mortality after ARDS diagnosis, and the cut-off value for the change in ΔP and MP to predict 30-day mortality was identified according to the ROC curve. Statistical significance was set at *p* < 0.05.

## 3. Results

**Figure 1:**
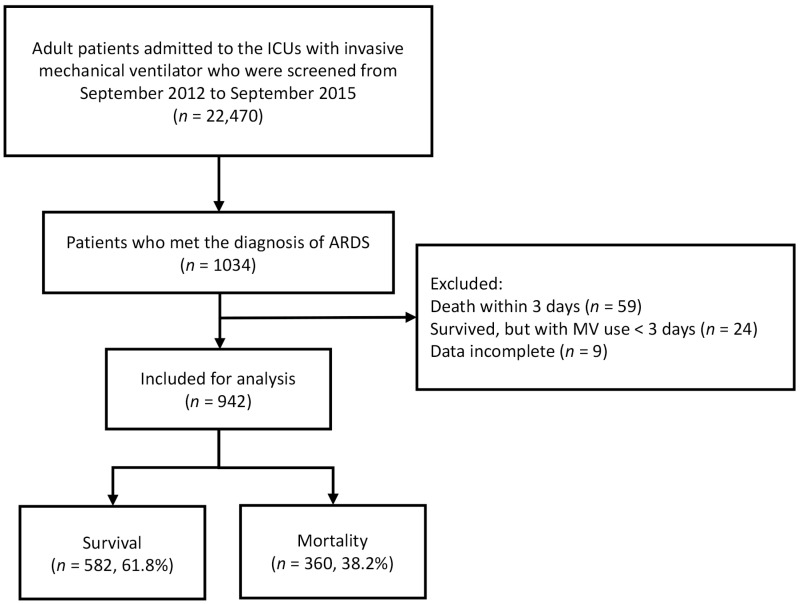
Flow chart of patient selection, enrollment, and exclusion. Abbreviations: ICUs = intensive care units; ARDS = acute respiratory distress syndrome.

Altogether, 22,470 patients were screened and 1034 patients had ARDS (Figure 1). Among these, 942 patients were enrolled in this study, and 92 patients were excluded. Altogether, 360 patients died and 582 patients survived within 30 days of ARDS diagnosis. Table 1 shows the baseline clinical characteristics and ventilator parameters of patients with ARDS and the comparison of these parameters between the survivors and non-survivors. The survivor group was of a lower age, and had a lower Charlson Comorbidity Index, APACHE II score, SOFA score, ΔP, change in ΔP, and MP than the non-survivor group did. The non-survivor group had a lower incidence of trauma than the survivor group did. There were no differences in gender and the severity of ARDS between the survivors and non-survivors. The lung injury scores, PaO_2_/FiO_2_ ratios, and the *V*_T_, PEEP, RR, and MP values were similar between the survivors and non-survivors.

**Figure 2:**
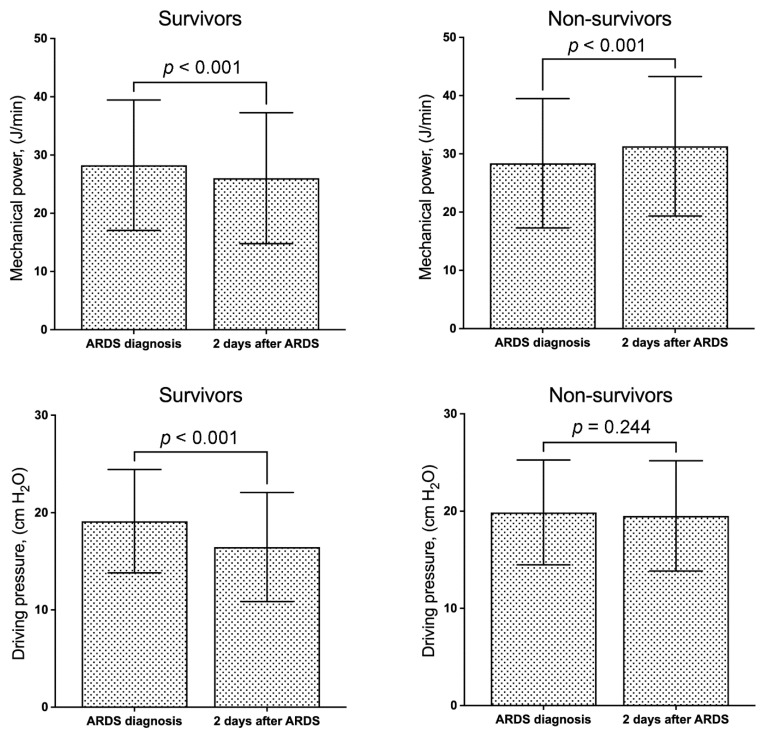
Column chart of mechanical power and driving pressure (mean ± 1 standard deviation) between survivors and non-survivors on the day of acute respiratory distress syndrome (ARDS) diagnosis and 2 days after ARDS diagnosis.

Compared with the MP and ΔP upon ARDS diagnosis, the MP and ΔP significantly decreased after 2 days of ARDS diagnosis in survivors (Figure 2). In non-survivors, MP significantly increased after 2 days of ARDS diagnosis but ΔP did not. According to the binary logistic regression model, the variables independently associated with 30-day mortality included age (odds ratio (OR), 1.012; 95% confidence interval (CI), 1.003–1.022), SOFA score (OR, 1.144; 95% CI, 1.086–1.206), trauma (OR, 0.172; 95% CI, 0.035–0.838), ΔP (OR, 1.077; 95% CI, 1.044–1.111), change in ΔP (OR, 1.087; 95% CI, 1.054–1.120), and change in MP (OR, 1.018; 95% CI, 1.006–1.029) (Table 2). Age, SOFA scores, ΔP, change in ΔP, and change in MP were positively correlated with 30-day mortality, while trauma was negatively correlated with it. The Charison Comorbidity Index and APACHE II score were not associated with 30-day mortality.

**Figure 3:**
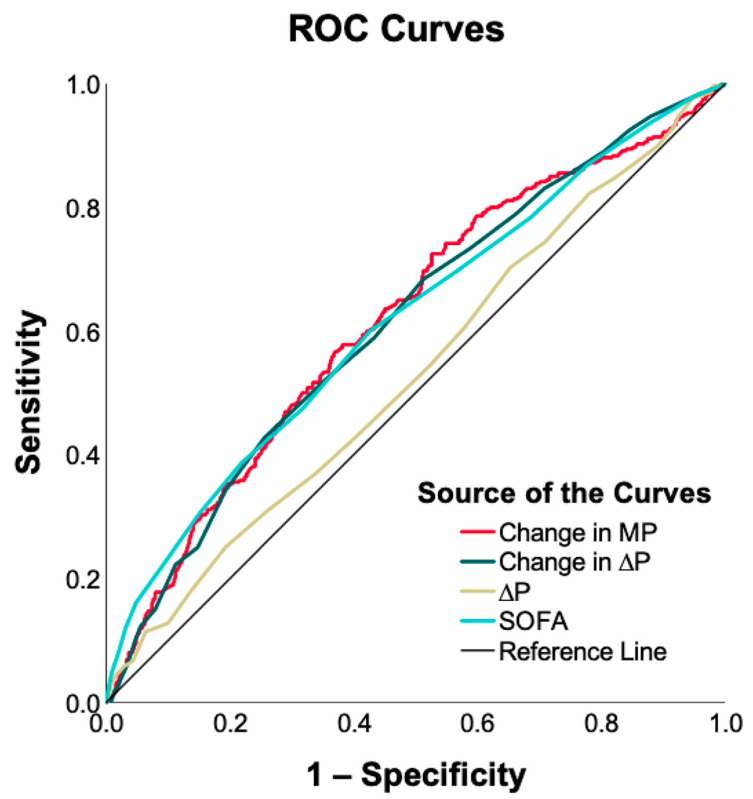
Receiver operating characteristic (ROC) curves of change in mechanical power (MP), change in driving pressure (ΔP), ΔP, and Sequential Organ Failure Assessment (SOFA) scores for 30-day mortality in patients with acute respiratory distress syndrome (ARDS). The areas under the ROC curve (AUROCs) were calculated. The AUROCs for change in MP, change in ΔP, ΔP, and SOFA scores were 0.620 (95% confidence interval (CI), 0.583–0.657; *p* < 0.001), 0.616 (95% CI, 0.579–0.653; *p* < 0.001), 0.532 (95% CI, 0.494–0.570; *p* = 0.096), and 0.617 (95% CI, 0.580–0.654; *p* < 0.001), respectively.

The AUROCs for the change in MP, change in ΔP, ΔP, and SOFA score were 0.620 (95% CI, 0.583–0.657; *p* < 0.001), 0.616 (95% CI, 0.579–0.653; *p* < 0.001), 0.532 (95% CI, 0.494–0.570; *p* = 0.096), and 0.617 (95% CI, 0.580–0.654; *p* < 0.001), respectively (Figure 3). The changes in MP and ΔP had similar predictive values for 30-day mortality. Changes in MP, changes in ΔP, and SOFA scores were always superior to ΔP in terms of the accuracy of predicting 30-day mortality.

**Figure 4:**
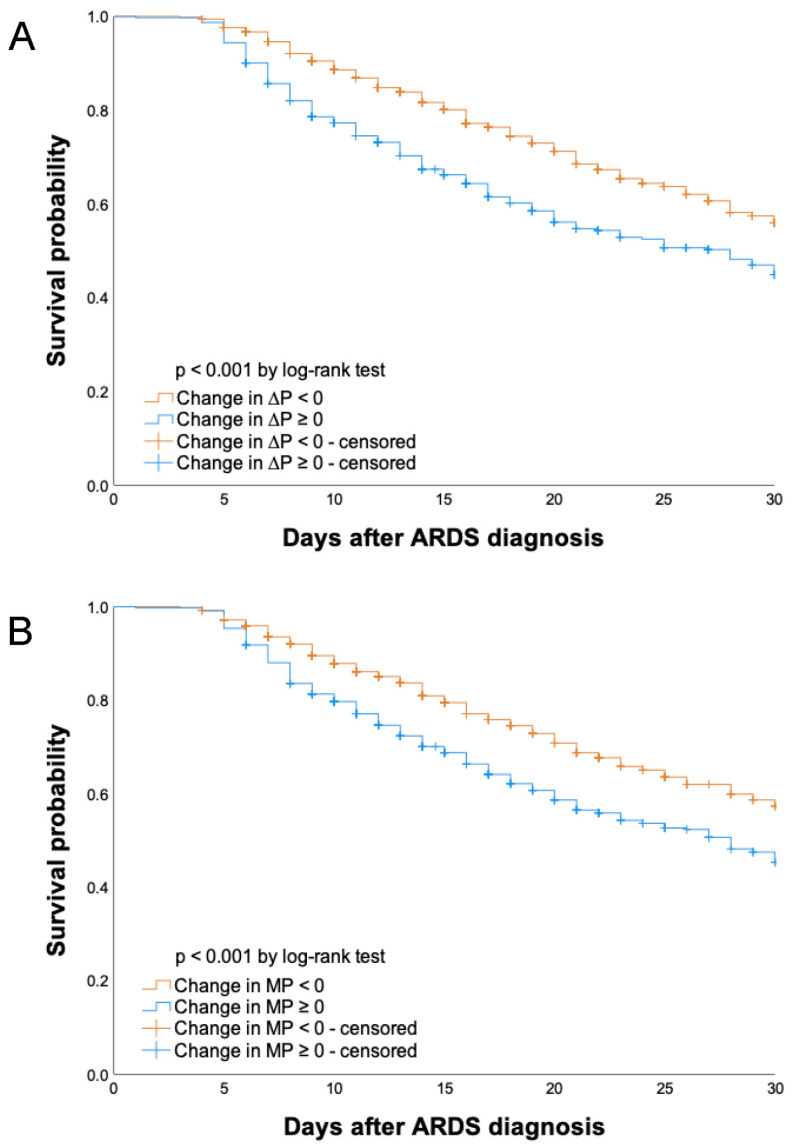
Kaplan–Meier graphs of 30-day cumulative survival in patients according to (**A**) changes in driving pressure (ΔP) and (**B**) changes in mechanical power (MP). Abbreviations: ARDS, acute respiratory distress syndrome.

To evaluate the changes in ΔP and MP, patients were divided into two groups according to serial decrement (changes in ΔP and MP < 0) and increment (changes in ΔP and MP ≥ 0). Thirty-day survival differed significantly between these groups (log-rank test, *p* < 0.001) (Figure 4). The 30-day survival rate in the serial decrement group was significantly higher than that in the serial increment group, regardless of whether ΔP or MP was used.

## 4. Discussion

According to the multivariate regression analysis, ΔP on the day of ARDS diagnosis and the changes in ΔP and MP were independently associated with 30-day mortality. The AUROCs of changes in ΔP and MP were higher than that of ΔP. A change in MP did not exhibit a better predictive value for 30-day mortality than changes in ΔP did. Since the calculation of changes in ΔP is easier than that of changes in MP, the former may be the first choice among all respiratory parameters to evaluate survival in patients with ARDS.

Our study is the first to report that changes in ΔP and changes in MP had superior predictive value for 30-day mortality when compared with ΔP on the day of ARDS diagnosis. This result was similar to that reported in Chang’s study, which showed that patients with ARDS had the lowest survival in the group with serial increments of ΔP [6]. Moreover, a change in ΔP was also positively correlated with the plasma concentration of interleukin-6 and the soluble receptor for advanced glycation end-products [24], indicating the greater severity of VILI with higher ΔP. Decrements in MP and ΔP 2 days after ARDS diagnosis suggest better survival during mechanical ventilation in patients with ARDS.

Kaplan–Meier graphs of cumulative survival in ventilated ARDS patients with pneumonia did not show a difference between the groups with high (≥27 J/min) and low (<27 J/min) MP [23]. This finding is consistent with our result that MP on the day of ARDS diagnosis was similar between survivors and non-survivors. However, initial MP might play the role of predicting mortality in critically ill patients with mild ARDS or non-ARDS. Ventilated non-ARDS patients with MPs of < 27 J/min on day 1 show higher survival rates [23]. In a database study from MIMIC–III and eICU, the best cutoff of MP found in an ROC analysis for in-hospital mortality was 19 J/min with poor predictive power [15]. The AUROC was 0.521, with 48% sensitivity and 56% specificity. In this database study, patients had mild ARDS or were non-ARDS patients with mean PaO_2_/FiO_2_ = 255 and 211 mmHg in MIMIC-III and eICU, respectively. Another probable cause of the different results between this study and the database study was the use of different equations for VCV and PCV.

Changes in MP showed differences between survivors and non-survivors in this large-scale cohort study. Since MP including ventilation-tidal volume, ΔP, flow, resistance, RR, and PEEP was treated as a unique physical energy delivered into the lung, this finding implied that the decreased need for MP to maintain adequate SpO_2_ or PaO_2_ levels after 2 days of ARDS diagnosis led to better disease control in the lung and resulted in lower mortality. It remains unclear whether MP is a predictor or a cause of lung injury. Further studies are required to elucidate this issue.

In the present study, ΔP on the day of ARDS diagnosis was an independent factor associated with 30-day mortality in patients with ARDS. However, the AUROC was 0.532, suggesting that there was no discrimination in ΔP between survivors and non-survivors. On the other hand, the change in ΔP (AUROC = 0.616) had a similar predictive value to that in SOFA, a widely used severity score. After combining the SOFA score and change in ΔP into one score, the predictive value did not increase significantly (AUROC = 0.659). Based on our results, the change in ΔP alone to predict patient survival was not inferior to the severity score and MP, with easy calculation and no need for blood sampling in clinical practice.

A personalized mechanical ventilation setting for patients with ARDS may improve respiratory parameters and outcomes. The most important recommendation for providing lung-protective ventilation in ventilated patients with ARDS is low *V*_T_ ventilation [25]. Esophageal pressure monitoring allows the estimation of transpulmonary pressure and assists with individual PEEP settings. However, its use requires technical skill and correct physiological interpretation for bedside clinical applications [26]. In 2015, a retrospective analysis reported that ΔP showed a stronger association with 60-day mortality than tidal volume did in patients with ARDS [5]. In 2016, a new concept of safe mechanical ventilation using MP was introduced [27]. MP was independently and positively associated with 28-day mortality and had a better predictive value than ΔP did in ventilated patients with pneumonia [23]. However, in an animal model of ARDS, high *V*_T_ resulted in VILI even at low mechanical power [28]. MP considers ventilatory parameters collectively in the optimization of ventilation settings, but further studies are necessary to assess its clinical relevance.

The present study has some limitations. This was a single-center trial without patients from another hospital. Multi-center studies are required to confirm our results. The mean *V*_T_ in the present study population was 8.4 mL/kg PBW on the day of ARDS diagnosis. This was relatively high and did not fit the low *V*_T_ ventilation strategy. This setting might have influenced our results to a certain degree. The adverse events were not recorded. Shock has been considered a strong risk factor for mortality [23,29,30]. It is unknown whether the changes in MV and ΔP were confounded by shock.

## 5. Conclusions

Our findings imply that changes in ΔP and MP are associated with 30-day mortality in ventilated ARDS patients. Incremental MP had a similar predictive value for 30-day mortality when compared with incremental ΔP. Since calculating the change in ΔP is easy for respiratory therapists in clinical practice, it may be used to predict mortality in patients with ARDS through the continuous monitoring of ΔP.

## Figures and Tables

**Table 1:** Baseline clinical characteristics and ventilator parameters in patients with ARDS and comparison of these parameters between survivors and non-survivors.

Characteristics	Total Patients (*n* = 942)	Survivors (*n* = 582)	Non-Survivors (*n* = 360)	*p*-Value
Age (years) *	63.1 ± 16.2	62.3 ± 16.8	64.4 ± 15.0	0.040
Gender, *n* (%)				0.605
Male	653 (69.3)	407 (69.9)	246 (68.3)	
Female	289 (30.7)	175 (30.1)	114 (31.7)	
Charlson Comorbidity Index *	2.6 ± 2.2	2.4 ± 2.1	3.0 ± 2.4	<0.001
APACHE II score *	23.2 ± 7.1	22.4 ± 7.1	24.5 ± 6.7	<0.001
SOFA score *	9.8 ± 3.4	9.2 ± 3.1	10.7 ± 3.7	<0.001
Lung injury score *	2.9 ± 0.5	2.9 ± 0.5	2.9 ± 0.5	0.066
Severity of ARDS, *n* (%)				0.155
Mild	214 (22.7)	143 (24.6)	71 (19.7)	
Moderate	377 (40.0)	233 (40.0)	144 (40.0)	
Severe	351 (37.3)	206 (35.4)	145 (40.3)	
Causes of ARDS, *n* (%)				0.020
Pneumonia	670 (71.1)	415 (71.3)	255 (70.8)	
Sepsis	127 (13.5)	70 (12.0)	57 (15.8)	
Aspiration	50 (5.3)	32 (5.5)	18 (5.0)	
Post-surgery	11 (1.2)	9 (1.6)	2 (0.6)	
Trauma	23 (2.4)	21 (3.6)	2 (0.6)	
Others	61 (6.5)	35 (6.0)	26 (7.2)	
PaO_2_/FiO_2_ (mm Hg) *	141.1 ± 74.3	142.2 ± 72.2	136.8 ± 70.6	0.261
ECMO support	57 (6.1)	41 (7.0)	16 (4.4)	0.122
Ventilator parameters *				
Tidal volume (mL/kg PBW)	8.4 ± 2.6	8.4 ± 2.6	8.4 ± 2.5	0.862
PEEP (cm H_2_O)	9.9 ± 2.1	9.9 ± 2.2	9.8 ± 2.0	0.728
Driving pressure (cm H_2_O)	19.4 ± 5.4	19.1 ± 5.3	19.9 ± 5.4	0.038
Respiratory rate (/min)	21.5 ± 5.9	21.6 ± 6.2	21.3 ± 5.3	0.411
Mechanical power (J/min)	28.3 ± 11.2	28.2 ± 11.2	28.4 ± 11.1	0.846
Ventilator parameters after 2 days *				
Tidal volume (mL/kg PBW)	8.3 ± 2.8	8.4 ± 3.0	8.3 ± 2.5	0.594
PEEP (cm H_2_O)	10.8 ± 2.6	10.5 ± 2.6	11.2 ± 2.6	<0.001
Driving pressure (cm H_2_O)	17.6 ± 5.8	16.5 ± 5.6	19.5 ± 5.7	<0.001
Respiratory rate (/min)	22.1 ± 5.6	21.4 ± 5.7	23.1 ± 5.3	<0.001
Mechanical power (J/min)	28.0 ± 11.8	26.0 ± 11.2	31.3 ± 12.0	<0.001
Change in driving pressure (cm H_2_O) *	−1.8 ± 6.0	−2.7 ± 5.9	−0.4 ± 5.8	<0.001
Change in mechanical power (J/min) *	−0.2 ± 13.9	−2.2 ± 13.5	2.9 ± 14.0	<0.001

Abbreviations: ARDS, acute respiratory distress syndrome; APACHE, Acute Physiology and Chronic Health Evaluation; SOFA, Sequential Organ Failure Assessment; PaO_2_, partial pressure of oxygen; FiO_2_, fraction of inspired oxygen; ECMO, Extracorporeal Membrane Oxygenation; PEEP, positive end-expiratory pressure. * Data are presented as mean ± standard deviation.

**Table 2:** Binary logistic regression to analyze the independent factors associated with 30-day mortality.

Variables	Univariate OR (95% CI)	*p*-Value	Multivariate OR (95% CI)	*p*-Value
Age	1.008 (1.000–1.017)	0.046	1.012 (1.003–1.022)	0.014
Charlson Comorbidity Index	1.134 (1.070–1.203)	<0.001	1.056 (0.990–1.127)	0.099
APACHE II score	1.042 (1.023–1.062)	<0.001	0.995 (0.970–1.020)	0.668
SOFA score	1.142 (1.097–1.189)	<0.001	1.144 (1.086–1.206)	<0.001
Trauma	0.128 (0.028–0.596)	0.009	0.172 (0.035–0.839)	0.029
Driving pressure	1.027 (1.002–1.052)	0.038	1.077 (1.044–1.111)	<0.001
Change in driving pressure	1.069 (1.044–1.094)	<0.001	1.087 (1.054–1.120)	<0.001
Change in mechanical power	1.028 (1.018–1.039)	<0.001	1.018 (1.006–1.029)	0.002

Abbreviations: OR, odds ratio; CI, confidence interval; APACHE, Acute Physiology and Chronic Health Evaluation; SOFA, Sequential Organ Failure Assessment.

## Data Availability

The datasets generated for this study can be obtained upon request from the corresponding author.
